# Use of a Biofeedback Breathing App to Augment Poststress Physiological Recovery: Randomized Pilot Study

**DOI:** 10.2196/12227

**Published:** 2019-01-11

**Authors:** David Plans, Davide Morelli, Stefan Sütterlin, Lucie Ollis, Georgia Derbyshire, Mark Cropley

**Affiliations:** 1 BioBeats Group LTD London United Kingdom; 2 Department of Experimental Psychology University of Oxford Oxford United Kingdom; 3 Department of Engineering Science Institute of Biomedical Engineering University of Oxford Oxford United Kingdom; 4 Faculty for Health and Welfare Sciences Østfold University College Østfold Norway; 5 Division of Clinical Neuroscience Oslo University Hospital Oslo Norway; 6 Faculty of Health and Medical Sciences School of Psychology University of Surrey Guildford United Kingdom

**Keywords:** biofeedback, breathing, heart rate variability, recovery, rumination, stress

## Abstract

**Background:**

The speed of physiological recovery from stress may be a marker for cardiovascular disease risk. Stress management programs that incorporate guided breathing have been shown to moderate the stress response and augment recovery.

**Objective:**

The aim of this study was to examine the effectiveness of an app-based brief relaxation intervention (BioBase) for facilitating physiological recovery in individuals exposed to a brief psychological stressor.

**Methods:**

A total of 75 participants (44 women) completed a stressor speech task and were randomly assigned to one of three conditions: control, rumination, or an app-based relaxation breathing (BioBase) conditions. Heart rate variability (HRV) was assessed as a measure of autonomic function at baseline (6 min), during stress (6 min), and during recovery (6 min).

**Results:**

There was a significant increase in subjective stress following stress exposure, but the ratings returned to baseline after recovery in all three groups. In addition, there was a significant decrease in vagally mediated HRV in the poststress period. During recovery, the root mean square of successive differences (*P*<.001), the percentage of successive interbeat (RR) intervals that differ by >50 ms (pNN50; *P*<.001), and high-frequency (*P*<.02) HRV were significantly higher in the BioBase breathing condition than the rumination and control conditions. There was no difference in HRV values between the rumination and control conditions during recovery.

**Conclusions:**

App-based relaxed breathing interventions could be effective in reducing cardiovascular disease risk. These results provide additional utility of biofeedback breathing in augmenting physiological recovery from psychological stress.

## Introduction

Cardiovascular disease is one of the leading causes of premature death and disability in most westernized countries and accounts for approximately 30% of deaths worldwide [1]. Its risk factors include genetic and congenital defects as well as behavioral factors such as diet, exercise, and smoking [2]. Another major risk factor for cardiac disease is mental stress [3,4]; mental stressors are known to cause physiological and psychological changes due to the activation of the sympathetic nervous system and parasympathetic withdrawal [5]. These changes include increased heart rate; respiration; and production of various biological stress markers including adrenaline, cortisol, and alpha-amylase. Prolonged stress exposure or delayed recovery from stress responses (ie, how quickly an individual returns to a specified resting baseline), is thought to contribute to cardiovascular disease risk, as it causes wear and tear of the cardiovascular system [6]. Growing research evidence suggests that recovery from stress may be more predictive of cardiovascular health than cardiovascular reactivity to the stressor itself [7-9].

Stress is a part of life and cannot be completely avoided. Therefore, it is crucial to identify and develop techniques that can augment recovery from stress in order to prevent the development of chronic health problems such as cardiovascular disease. One effective method for the management of stress is the use of biofeedback systems [10]. Biofeedback is a mind-body self-regulation practice, wherein individuals learn how to regulate their thoughts, feelings, and behavior to modify their physiology through continuous physiological feedback. For example, by instantaneously translating heart rate onto a visual display, an individual can quickly learn how slow breathing can be used effectively to reduce the heart rate. Biofeedback training has beneficial effects on many symptoms of stress, including anxiety and depression, and improves the overall health [11].

There are many different physiological stress markers such as elevated blood pressure, heart rate, and cortisol levels, but this study focused on heart rate variability (HRV). Changes in HRV have been shown to reflect levels of stress induced by a mental stressor [12,13]. In addition, HRV is a marker of autonomic dysfunction, and a reduced HRV has been associated with an increased risk of cardiovascular disease and mortality [14-16]. HRV is considered a nonintrusive, objective, discrete measure of vagally mediated cardiac regulation. Vagally mediated HRV refers to the beat-to-beat variability in heart rate that is controlled by the parasympathetic nervous system via the vagus nerve. Regulating emotions is a key life skill, and emotional changes appear to occur simultaneously with changes in HRV. Thus, HRV may be a marker of emotional regulation, which is activated when individuals are under mental stress [13,17-20]. The heart rate increases slightly during inspiration and decreases slightly during expiration; slow breathing evokes respiratory sinus arrhythmia, through which the heart rate and breathing synchronize, thereby increasing oscillations within the heart. Respiratory sinus arrhythmia is used as an index of cardiac vagal tone (referring to the activity of the vagus nerve) and contributes to HRV. Deep, slow diaphragmatic breathing stimulates the vagus nerve, thus improving HRV and reducing stress responses. By slowing down breathing, an individual can learn to control his/her heart rate and, ultimately, the HRV by increasing parasympathetic activity [21].

HRV biofeedback has resurged in recent years, and biofeedback interventions have been effective after an emotional disturbance [22]. In addition, interventions that include breathing exercises have been shown to reduce stress responsivity in both the laboratory and real world [23-25], although these interventions are time consuming and typically developed as part of a suite. For example, the body scan breathing exercise, which forms the basis of most mindfulness programs, requires repeated practice with typical sessions of ≥30 min [25], whereas some studies that incorporate breathing routines require attendance in 10 treatment sessions, each lasting 90 min [24]. Such interventions, although effective, require investment of a lot of time from users, and many people feel time starved. Therefore, easy-to-use parsimonious interventions that mitigate the effects of stress are clearly advantageous. It is now possible for mobile health interventions to incorporate biofeedback breathing exercises in order to help people regulate their emotions during acute periods of stress.

Technology has paved the way and provided an easy and accessible method to engage with and monitor stress levels. Breathing techniques and biofeedback can now be easily combined to create an effective coping tool for stress management. Mobile technology can be used to effectively deliver stress-management techniques [26,27]. Through consumer-grade sensors, mobile phones provide an excellent avenue for collecting continuous biometric data such as those on heart rate and HRV. Such applications allow people to access their HRV biofeedback and modify their breathing, thereby gaining better control over stress [28]. Deep-breathing mobile phone apps are easy to use and inexpensive and have the potential to reach a large population; thus, they have far-reaching implications for long-term health.

This study aimed to examine a commercial breathing app called BioBase [29] as a potential intervention to reduce poststress activation. The BioBase program reduces stress responsivity by coaching users through Papworth breathing exercises, both audibly and visually. The regulation of breathing triggers a vagal response to aid parasympathetic activation, leading to relaxation.

To test the efficacy of the BioBase app, we subjected participants to a standard psychological stressor task and then randomly assigned them to one of three conditions: the BioBase breathing app, rumination, and control conditions. The rationale for using a rumination condition was amplification of possible differences in HRV between the three conditions. We hypothesized that compared to the rumination and control conditions, the Biobase breathing app would enhance recovery following exposure to a stressful speech task.

## Methods

### Participants

A total of 75 participants were recruited to the study, including 44 (59%) women and 31 (41%) men, with an average age of 23.5 years (SD 6.39; range, 18-55 years). The sample predominantly identified themselves as white British (n=55, 73%) and the remainder, as Asian (n=8, 11%), white other (n=4, 5%), African (n=2, 3%), black British (n=1, 1%), Greek (n=1, 1%), Hispanic (n=1, 1%), mixed Hispanic (n=1, 1%), and Mauritian (n=1, 1%). All participants received a £15 gift voucher for their time. The study was approved by the ethics committee of the University of Surrey, UK, and written informed consent was obtained from all participants (identifier: 1190-PSY-16).

### Speech Task

During the stressor phase of the study, participants listened to a 2-min audio recording that described a situation where they had been falsely accused of stealing a purse and had to defend themselves to the police; this stressor has been used in previous studies (eg, [30]). The participants were instructed to imagine themselves in the scenario and were given 2 min to mentally think and prepare a statement for their defense in front of a video recorder. The recording stated that their speech would be evaluated and marked by the experts for fluency and confidence. This aimed to instill a social evaluative threat that has been shown to increase stress and rumination and induce physiological reactivity [31,32]. However, in reality, the speeches were not evaluated, as the performance ratings were not relevant to this study.

### BioBase App

Participants in the BioBase condition were guided through a clinically validated version of the Papworth-Benson breathing exercise [33]. Originally developed at Papworth Hospital in Cambridgeshire, UK, more than 5 decades ago, this breathing method focuses on diaphragmatic breathing to use the full lung capacity and slow down breathing as well as trigger vagus nerve stimulation and consequently, the relaxation response. Through the process of photoplethysmogram and the use of the camera and flash on the mobile phone, the app measured changes in the user’s heart rate during a guided breathing exercise and displayed their heart wave on the mobile phone screen in real time. Participants in the control and rumination conditions used a modified version of the Biobase app during the intervention period, which measured their heart rate, but did not guide them through the breathing exercise.

### Self-report measures

#### Thoughts Questionnaire

Following a stress-exposure task, individuals may spontaneously ruminate [34] and therefore confound the results. To assess poststress rumination, we used a modified version of the Thoughts Questionnaire [35]. Items were rated on a 5-point scale, from 0 (*never*) to 4 (*very often*), on how often they thought about each item during the intervention period (eg, I made a fool of myself and how awkward I felt). The Thoughts Questionnaire has good internal consistency [36] and has been used in many previous studies to assess postevent rumination following the delivery of a speech task [32,37,38]. Cronbach’s alpha for this measure was .81 in the present study.

#### Heart Rate Variability

HRV was captured during baseline, the speech task stressor, and poststress recovery by using a Biopac ECG100C amplifier (Biopac Systems Inc, Santa Barbara, CA). The electrocardiogram was recorded with a sampling rate of 1000 Hz.

### Procedure

After entering the laboratory, participants were asked to read the information sheet explaining the study. After providing written consent, the participants completed a demographic questionnaire. They were subsequently fitted with electrodes and acclimatized to the Biopac amplifier. The Biopac amplifier recorded heart data continuously throughout the experiment. All participants then relaxed for 6 min while the experimenter left the room to minimize distraction. On resuming the exercise, the participants rated their level of stress using a 7-point visual analogue scale (1, *very little pressure* to 7, *extreme pressure*). Thereafter, the participants listened to the audio stressor recording for 2 min, as described above in the Speech Task section, and were given 2 min to prepare a statement for their defense. They then presented their statement for 3 min in front of the experimenter and a video camera. Subsequently, the participants rated their level of stress using the 7-point stress scale. Following the speech task, participants were randomly assigned using a random number-generator program [39] to one of the three groups (control, BioBase, or rumination) for 6 min. As this study was not a preregistered randomized controlled trial, it was considered a pilot study.

Participants assigned to the breathing condition used the BioBase app on the mobile phone and followed the guided breathing instructions. Individuals in the rumination condition were asked to think and reflect about their speech and focus on performance-based thoughts and were guided during this task by a modified version of the Thoughts Questionnaire [35]. Participants in the control condition were asked to sit passively. At the end of the intervention period, participants rated their level of stress. All participants then completed the original Thoughts Questionnaire [35] to measure the actual levels of rumination during the intervention phase. Finally, participants were disconnected from the equipment, fully debriefed, and given a gift voucher.

### Data Analysis

All data were screened for measurement artifacts using the ARTiiFACT software [40] by one of the authors (SS) who was blinded to the conditions. Artifacts were identified using the algorithm developed by Berntson and colleagues [41], and a distribution-based threshold value calculated individually for each participant. Flagged beat intervals were visually checked and, if confirmed as artifacts, deleted and substituted by means of cubic spline interpolation of neighboring intervals. HRV analysis is typically performed in time or frequency domains. The time-domain measure root mean square successive difference (RMSSD) is considered to indicate vagally mediated HRV; values < 50 ms are considered unhealthy, values > 100 are considered healthy, and values in between indicate risk. The frequency-domain analysis high frequency (HF), which is considered to depict parasympathetic regulation, was also calculated for each person. Measurements and analyses followed established guidelines [42]. For the analysis, we grouped the HRV data into baseline, stressor, and intervention groups and analyzed 6-min segments, which are sufficient for HRV analysis [43]. Following the standard procedure, data were screened, outliers above and below 3 SDs were excluded, and data were transformed where necessary.

## Results

There was no significant difference between the groups with regard to demographic variables (gender, physical fitness, smoking status, general stress, and age), any baseline measures of subjective stress, or HRV. Ratings of perceived stress significantly increased from baseline (mean=2.01, SD 0.97) following completion of the stressor (mean=5.05, SD 1.37; *t*=18.68, *P*<.001), demonstrating successful experimental manipulation. Perceived stress returned to baseline levels in all three groups at the end of the recovery period. From the baseline period to the stress period, there was a significant increase in the heart rate (mean=78.59, SD 10.90 to mean=88.10; SD 13.25; *t=* –8.60; *P*<.001) and a significant decrease in the RMSSD (mean=44.13, SD 22.84 to mean=40.09, SD 15.56; *t*=1.72; *P*=.04), percentage of successive interbeat (RR) intervals that differ by >50 ms (pNN50; mean=17.75, SD=15.56 to mean=13.46, SD=11.18; *t*=3.35, *P*<.001), and HF (mean=2.77, SD=0.42 to mean=2.67, SD=0.37; *t*=2.42; *P*=.01; one-tailed), demonstrating autonomic activation in response to stress. There were no main effects of interaction or group. However, there was a significant difference between groups (*F*_1,2_=10.46; *P<*.001) in poststress rumination: The rumination group reported greater rumination (mean=1.97, SD 0.58) than the BioBase (mean=1.44, SD 0.79) and control (mean=1.37, SD 0.43) groups. There was no significant difference between the BioBase and the control groups in terms of poststress ruminative thoughts. Thus, the intervention manipulation was successful.

### Cardiac Analysis

To examine the effects of the intervention on cardiac recovery in the poststress period, a between-subjects analysis of covariance for group (Biobase, rumination, and control) was conducted using the recovery measures as the outcome and the reactivity change score as a covariate. As individual differences in stress response could influence the findings, following previous research [9], we treated reactivity as a covariate to statistically remove the influence of reactivity on recovery. Apart from heart rate, a significant effect of group, with medium-to-large effect size, was found for RMSSD, pNN50, and HF. In addition, in all cases, the recovery rate was higher in the BioBase group than in the rumination and control groups. There were no significant differences between the rumination and control groups. Thus, individuals who used the breathing app demonstrated enhanced recovery from stress. The results of the analysis are presented in Table 1.

**Table 1:** Results of the data analysis. Values on each line with an astericks (*) or dagger (†) were not significantly different from one another.

Parameter	BioBase group, mean (SD)	Control group, mean (SD)	Rumination group, mean (SD)	F value	*P* value	Effect size, partial eta squared
Heart rate, bpm	74.02 (9.25)*	78.52 (9.32)*	79.53 (10.40)*	2.58	.08	.07
RMSSD^a^	65.9 (20.51)	39.88 (20.73)*	43.77 (27.46)*	9.25	.001	.22
pNN50^b^	30.14 (10.89)	16.26 (15.34)*	18.14 (15.34)*	9.17	.001	.21
High frequency^c^	2.97 (0.37)*	2.74 (0.39)*,†	2.65 (0.37)†	4.15	.02	.11

^a^RMSSD: root mean square successive difference.

^b^pNN50: percentage of successive inter-beat RR intervals that differ by >50 ms.

^c^Log transformed.

## Discussion

### Principal Findings

As stress is an unavoidable part of life and the workplace becomes increasingly pressured, providing fewer opportunities for recovery, it is important to identify easy-to-use practical aids that can help individuals unwind and recover after the stressful period. The main finding of this study was that physiological recovery following stress exposure was enhanced in individuals who were guided through the BioBase breathing condition. This is an interesting finding because higher HRV is associated with reduced cardiovascular risks and other health outcomes [18,20,44].

We examined the effects of rumination on recovery in this study. Cognitive rumination has been defined as repetitive and intrusive negative thinking about past stressor(s) [45], and rumination is one of the main contributing factors that disrupts poststress recovery [46,47]. We were successful in manipulating rumination in the laboratory; however, rumination in the present study was not associated with reduced HRV, as there was no significant difference in poststress recovery between the rumination and control groups (as compared to [48]), although both groups showed reduced HRV as compared to the BioBase group.

Although there was a difference between the groups with respect to physiological parameters during recovery, there was no difference in subjective stress, as the levels of perceived stress returned to baseline for all groups in the recovery period. This finding is intriguing and suggests that perceptions of and actual physiology are not always in accordance. Individuals may think they have recovered from the effects of stress but may be unaware of their actual physiological state. This may not be an issue in the short term, but a mismatch between one’s psychological and physiological state could lead to long-term health issues over time, especially in individuals who are exposed to high levels of stress or work in demanding jobs. Interventions aimed at increasing sensitivity to one’s internal body state—perhaps, by enhancing interoceptive awareness—are needed. Somatic, or interoceptive awareness, could be defined as one’s perceptual awareness of signals from the inside the body, such as heartbeats, breathing, bowel movements, and higher-order processes including beliefs and attitudes as well as emotions regarding those perceptions [49-51]. Interoceptive awareness can be measured and trained. Such training might rely on the fact that simply noticing feelings, particularly bodily sensations associated with particular feelings, can foster emotional regulation. This is an aspect of mindfulness training that has been previously explored in studies of meditation training on interoception [52,53]. In the future, studies should focus on the integration of multimodal systems such as digital screen-based devices (mobile phones) and multidimensional theoretical models of interoception that take into account measurement of autonomic nervous system disruption using HRV data in order to build multimodal, digital therapeutic systems that measure interoception as well as offer training experiences that might help manage anxiety-based disorders embedded in apps such as BioBase.

### Limitations

There are some caveats with this present study. Heart rate monitoring was stopped after 6 min; therefore, we do not know how long the actual effect on HRV lasted. It will be of interest for future studies to establish the benefits of guided breathing over time and examine whether the effect fades, stabilizes, or increases with repetition. Repeated use of the guided breathing app may help individuals who chronically experience stress to recover more quickly physiologically and improve their health outcomes and functioning. Although physical fitness has been associated with enhanced physiological recovery from stress [7,54], we did not assess the levels of physical fitness in this study, which could be perceived as a limitation. However, there was no significant difference between the groups in terms of self-reported physical activity, and therefore, it seems extremely unlikely that physical fitness could account for the difference between the conditions in HRV recovery. Nonetheless, future research should control for levels of fitness to completely rule out the possibility that fitness played a key role in our findings.

### Conclusions

Given the ubiquitous nature of stress and the documented evidence associating stress with cardiovascular disease and mortality, it is important to identify affordable and practical aids that help individuals unwind and recover after the stressful period. To our knowledge, this is the first study to examine the effects of the BioBase breathing app on HRV under controlled conditions and provide evidence for its efficacy in aiding poststress recovery. These findings also provide evidence of the utility of biofeedback breathing in augmenting physiological recovery and suggest that app-based breathing interventions are effective in reducing cardiovascular disease risk.

## Abbreviations

HF: high frequency
HRV: heart rate variability
M: mean
RMSSD: root mean square successive difference
